# Selinexor in combination with dexamethasone with or without bortezomib in heavily pretreated multiple myeloma: A case series

**DOI:** 10.1002/jha2.913

**Published:** 2024-08-06

**Authors:** Nini Aung, Margaret Bowers, Gillian Brearton, Andrew Charlton, Joanne Craig, Jonathan Cullis, Ray Dang, David Donaldson, Mary Drake, Rachel Hall, Elizabeth Parkins, Jane Tighe, Ceri Bygrave, Oonagh Sheehy

**Affiliations:** ^1^ North Tees and Hartlepool NHS Foundation Trust Stockton‐on‐Tees Hartlepool UK; ^2^ Ulster Hospital Southeastern Health and Social Care Trust Dundonald UK; ^3^ Clatterbridge Cancer Centre NHS Foundation Trust Liverpool UK; ^4^ The Newcastle upon Tyne Hospitals NHS Foundation Trust Newcastle Upon Tyne UK; ^5^ Raigmore Hospital Inverness UK; ^6^ Salisbury NHS Foundation Trust Salisbury UK; ^7^ South Tees Hospitals NHS Foundation Trust Middlesbrough UK; ^8^ Belfast Health and Social Care Trust Belfast UK; ^9^ University Hospitals Dorset UK; ^10^ Royal Cornwall Hospitals NHS Trust Truro UK; ^11^ NHS Grampian Aberdeen UK; ^12^ Cardiff and Vale UHB Cardiff UK

**Keywords:** case series, objective response rate, progression‐free survival, safety, selinexor, UK

## Abstract

This report describes the characteristics and outcomes of 18 heavily pretreated patients with multiple myeloma (MM) who were subsequently treated with selinexor. This is a case series of 18 patients with MM who were treated with selinexor and dexamethasone (Sd) or selinexor, bortezomib, and dexamethasone (SVd) in 12 hospitals in the UK between 2019 and 2021. Eight patients received Sd and 10 patients received SVd. Patients received a median of five prior treatment lines, including immunomodulatory agents in 94% and proteasome inhibitors in 94%. Ten patients (55%) had triple‐class refractory disease. Six of the 12 evaluable patients achieved ≥partial response. The median progression‐free survival was 5.6 months, which was higher with SVd (5.7 months) than with Sd (2.1 months). The results support a treatment benefit of selinexor in heavily pretreated patients and support the notion that selinexor may overcome resistance to prior therapies, with no new safety concerns arising.

## 1 INTRODUCTION

Multiple myeloma (MM) is a hematological malignancy characterized by clonal plasma cells in the bone marrow or a biopsy‐proven plasmacytoma and by hypercalcemia, renal insufficiency, anemia, and/or lytic bone lesions [1]. Current treatment options include, but are not limited to, autologous stem cell transplantation (ASCT), immunomodulatory agents (IMiDs), proteasome inhibitors (PIs), monoclonal antibodies, the exportin‐1 (XPO1) inhibitor, selinexor, and the antibody‒drug conjugate targeting B‐cell maturation antigen, belantamab mafodotin [2].

Therapeutic management of MM remains difficult, as patients’ disease invariably relapses, develops drug resistance, and becomes refractory to treatment [1]. Although there are several first‐ and second‐line therapeutic options, treating relapsed/refractory MM (RRMM) in patients who have received two or more prior lines of therapy is challenging [2]. For triple‐class refractory patients (i.e., patients whose disease is refractory to at least one PI, one IMiD, and daratumumab), the 2021 European Hematology Association‐European Society of Medical Oncology guidelines recommend selinexor‒dexamethasone (Sd) or belantamab mafodotin monotherapy as suitable therapeutic options [2].

By blocking XPO1, selinexor forces nuclear accumulation as well as activation of tumor suppressor proteins, inhibits nuclear factor κB, and reduces oncoprotein messenger RNA translation [3, 4]. Sd is approved by the United States Food and Drug and Administration (US FDA) and the European Medicines Agency (EMA) for adult patients with RRMM [3] who have received at least four prior therapies and whose disease is penta‐refractory. In addition, selinexor in combination with bortezomib and dexamethasone (SVd) is approved by the US FDA and the EMA for patients with MM with at least one prior therapy (SmPC). Herein, we describe the outcomes of 18 patients with MM treated with selinexor after failure of three or more prior treatment lines.

## 2 CASE SERIES PRESENTATION

This case series included 18 patients with MM who were treated with Sd (*n* = 8) or SVd (*n* = 10) in 12 hospitals in the UK between 2019 and 2021. At diagnosis, 11 of the 16 patients with known myeloma types had immunoglobulin G myeloma. The International Staging System classification at diagnosis was available for 12 patients; two patients were classified as stage I, three as stage II, and seven as stage III. Overall, the median age at diagnosis was 59.5 years (range 44‒72), which was similar in patients treated with Sd or SVd. Patients were treated with a median of five prior treatment lines (Sd: 5.5 and SVd: 5). Overall, nine patients had previously undergone ASCT. Previous therapies included at least one IMiD (lenalidomide and/or thalidomide and/or pomalidomide) in 94% and at least one PI (bortezomib and/or carfilzomib and/or ixazomib) in 94%. For 10 patients, the disease was triple‐class refractory, and for four patients, it was penta‐refractory (Table 1).

**TABLE 1:** Demographic and disease characteristics.

Characteristic	Sd (*N* = 8)	SVd (*N* = 10)	Total (*N* = 18)
Median age at start of treatment with selinexor (years) (range)	57.5 (53–72)	61.0 (44–72)^a^	59.5 (44–72)^a^
Gender, *n* (%)			
Male	4 (50)	4 (40)	8 (44)
Female	4 (50)	6 (60)	10 (56)
Type of MM, *n* (%)			
IgG	6 (75)	5 (50)	11 (61)
IgA	2 (25)	0	2 (11)
Light chain	0	2 (20)	2 (11)
Non‐secretory	0	1 (10)	1 (6)
Unknown	0	2 (20)	2 (11)
Prior lines of therapy, *n* (%)			
3‒5	4 (50)	6 (60)	10 (56)
6‒18	4 (50)	2 (20)	6 (33)
Unknown	0	2 (20)	2 (11)
Median no. of prior lines of therapy (range)	5.5 (3–8)	5 (4–8)	5 (3–8)
Previous ASCT, *n* (%)	5 (63)^b^	4 (40)^b^	9 (50)
Previous therapy, *n* (%)			
Anti‐CD38	5 (63)	5 (50)	10 (56)
At least one immunomodulatory agent	8 (100)	9 (90)	17 (94)
Lenalidomide	8 (100)	9 (90)	17 (94)
Pomalidomide	8 (100)	8 (80)	16 (89)
Thalidomide	7 (88)	5 (50)	12 (67)
At least one proteasome inhibitor	8 (100)	9 (90)	17 (94)
Bortezomib	8 (100)	9 (90)	17 (94)
Carfilzomib	3 (38)	3 (30)	6 (33)
Ixazomib	1 (13)	3 (30)	4 (22)
Triple‐class refractory^c^	6 (75)	4 (40)	10 (55)
Penta‐refractory	1 (13)	3 (30)	4 (22)
Unknown	0 (0)	1 (10)	1 (5)

Abbreviations: ASCT, autologous stem cell transplantation; Ig, immunoglobulin; MM, multiple myeloma; Sd, selinexor and dexamethasone; SVd, selinexor, bortezomib, and dexamethasone.

^a^ Missing age information (SVd, *n* = 2).

^b^ Missing ASCT information (Sd, *n* = 1; SVd, *n* = 3).

^c^ Triple‐class refractory patients include penta‐refractory patients.

Patients (*n* = 8) receiving the Sd regimen started selinexor at a dose of 80 mg twice weekly (BIW), but the dose could be adjusted to 100 mg once weekly (QW) or 60 mg BIW to mitigate adverse events (AEs). Similarly, 100 mg QW was the starting dose for patients (*n* = 10) receiving SVd, which could be further reduced to 80 or 60 mg QW. The best evaluable responses for patients (*n* = 6) treated with Sd were very good partial response (VGPR) in one patient, partial response (PR) in three patients, and minimal response in one patient (Table S1). The best evaluable responses in patients treated with SVd (*n* = 6) were complete response in one patient, PR in one patient, and stable disease in three patients. One patient was eligible for transplantation post‐SVd treatment, although the response was not known. The response was not evaluated for one patient who was unable to tolerate treatment and four patients discontinued treatment due to disease progression. Six out of the 12 patients (overall response rate [ORR] 50%) with available response assessment achieved a response ≥PR. Additionally, two patients treated with Sd and one with SVd discontinued treatment prior to completion of a full treatment cycle due to rapid deterioration from advanced disease at the start of treatment or due to AEs; one patient contracted COVID‐19 and discontinued treatment.

**FIGURE 1:**
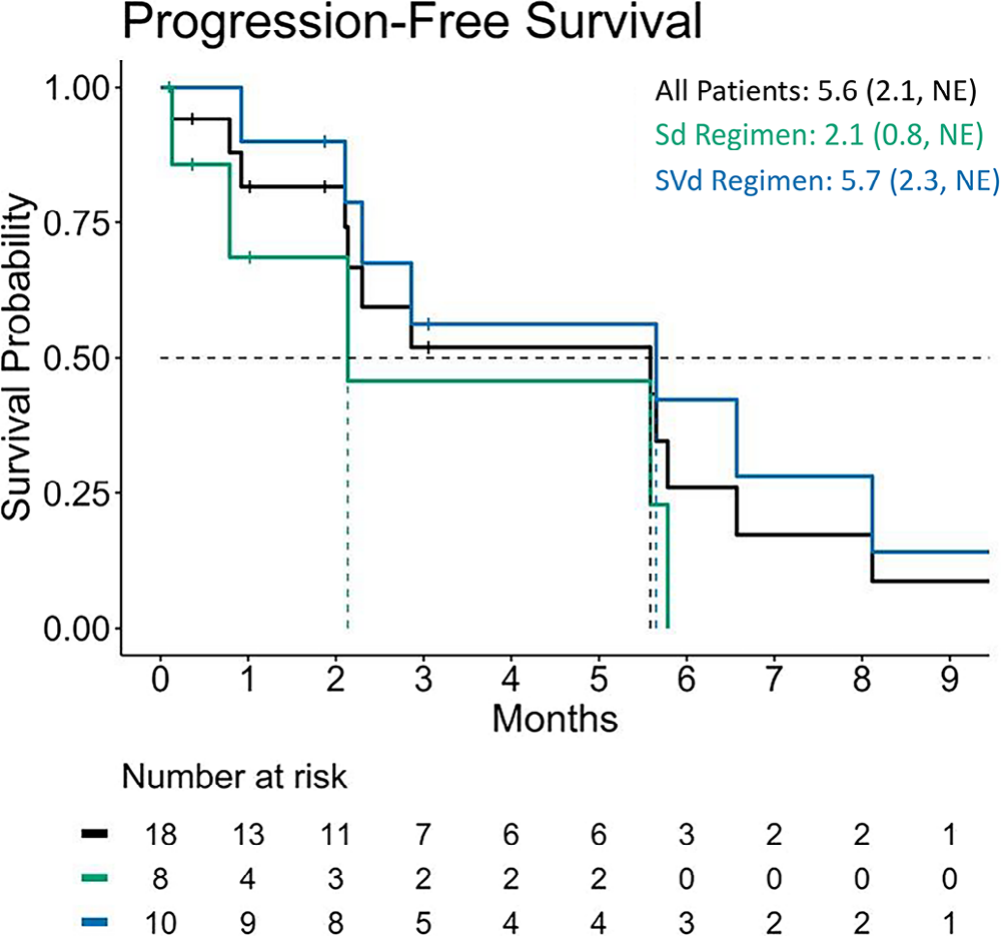
Progression‐free survival plot. In total of the 18 patients, 13 experienced an event of progression (*n* = 12) or death (*n* = 1), while the remaining patient events were censored. NE, not evaluable; Sd, selinexor and dexamethasone; SVd, selinexor, bortezomib, and dexamethasone.

Overall, of the 18 patients, 12 (four treated with Sd and eight with SVd) experienced disease progression, while one patient treated with Sd died due to lung infection unrelated to selinexor treatment. The remaining events in patients were censored for reasons provided in Table S2. The Kaplan‒Meier estimated median progression‐free survival (PFS) was 5.6 months, which was higher among patients treated with SVd (5.7 months) than among those treated with Sd (2.1 months) (Figure 1).

The most common hematological AEs were thrombocytopenia and neutropenia. The incidence of grade ≥3 thrombocytopenia was similar with Sd and SVd (40% and 50%, respectively). Grade ≥3 neutropenia was reported in 25% of the patients treated with Sd and in 0% of those treated with SVd. The most common non‐hematological AEs were nausea, vomiting, and hyponatremia. Grade ≥3 nausea, vomiting, and hyponatremia were reported for 25%, 0%, and 13% of the patients treated with Sd and for 20%, 20%, and 0% of those treated with SVd, respectively (Table S3).

## 3 DISCUSSION

In this case series of 18 patients who had received at least three prior treatment lines and the majority of whom were refractory to at least one IMiD and one PI, the overall PFS following selinexor‐based treatment was estimated to be 5.6 months, with 50% of the evaluable patients achieving a ≥PR. Among patients receiving Sd, the respective values were 2.1 months and 67%, whereas in the STORM trial, which enrolled patients who had received a median of seven prior treatment lines, the median PFS with Sd was 3.7 months and the ≥PR rate was 26% [3]. In the present case series, PFS was higher with SVd than with Sd (5.7 months vs. 2.1 months). The observed PFS with SVd was lower than that observed in the phase III BOSTON trial, which is to be expected since patients in this case series were more heavily pretreated [5].

Regarding other real‐world experiences with selinexor, in a case series of 13 patients with RRMM treated with SVd after having exhausted other treatment options, the ORR was 23% (all VGPRs) [6], whereas in another real‐world case series from Belgium, seven patients treated with SVd who had received a median of eight prior lines and the majority (86%) of whom were penta‐refractory the ORR was higher than that reported herein (71.4%) [7].

The value of any comparison of the effectiveness results of this case series to the effectiveness results from real‐world studies with other MM therapies in heavily pretreated patients is restricted by potential differences in patient characteristics, as well as by the fact that the small number of patients in the present work precludes meaningful analysis of factors that may have impacted response data. Even so, effectiveness findings observed are similar to those in a retrospective review of data from 75 patients previously treated with a median of four prior treatment lines who received a combination of panobinostat, bortezomib, and dexamethasone (ORR: 47% and median PFS: 3.5 months) [8]. In both the case series with panobinostat and the present study, PFS was longer in the subgroup of patients who achieved a ≥PR, reaching 6.2 months in both reports [8]. In addition, the ORR and PFS herein are within the range of those reported in real‐world studies among patients with double‐refractory MM treated with daratumumab monotherapy. Specifically, the reported ORRs were 28.6% [9], 42.1% [10], and 46% [11], and the estimated PFSs were 4.1 [9], 6 [10], and 2.7 months, respectively [11].

The observed AEs of selinexor are in alignment with the known safety profile. The most common and severe AEs were thrombocytopenia and gastrointestinal toxicity, whereas fatigue, a common AE with selinexor, was recorded for one patient. These findings suggest that, as with every first‐in‐class drug, the initial challenge physicians face to manage AEs is dissipated as they gain familiarity with the safety profile, and follow guidelines for the management of the drug‐specific AEs, as is the case for selinexor [12, 13].

The major limitations of our work include the small sample size, its retrospective nature, and the large number of non‐evaluable patients, which limited results interpretation. Despite these limitations, this study presents the first collective work of cases treated with selinexor across the UK in multiple clinics and therefore reflects differences in medical practice paradigms.

In summary, the results of the present study are encouraging in terms of effectiveness of SVd and Sd in heavily pretreated patients, with ORR and PFS supporting a treatment benefit and the notion that selinexor may overcome resistance to prior therapies, with no new safety concerns arising.

## AUTHOR CONTRIBUTIONS

All authors contributed data to the study, reviewed the initial manuscript, and approved the final draft. Oonagh Sheehy contributed to the collection and analysis of data and the writing of the manuscript.

## CONFLICT OF INTEREST STATEMENT

The authors declare no conflicts of interest.

## FUNDING INFORMATION

The authors received no specific funding for this work.

## ETHICS STATEMENT

The authors have confirmed ethical approval statement is not needed for this submission.

## PATIENT CONSENT STATEMENT

Retrospective analysis of patient data in a non‐identifying manner was obtained from the Ethics Committee of United Kingdom Research and Innovation.

## CLINICAL TRIAL REGISTRATION

The authors have confirmed clinical trial registration is not needed for this submission.

## Supporting information

Supporting Information

## Data Availability

The data that support the findings of this study are available from the corresponding author upon reasonable request.
