# Supplementary material for: Selinexor in combination with dexamethasone with or without bortezomib in heavily pretreated multiple myeloma: A case series
Source: EJHaem. 2024 Aug 6;5(5):987–91. doi: 10.1002/jha2.913 (PMC11474306; doi:10.1002/jha2.913)
Supplement: Supplementary file 1 — Supporting Information [file JHA2-5-987-s001.docx]

**Supplementary Tables**

**Table S1. Response to Treatment**

| **Characteristic** | **Sd**  **(N=8)** | **SVd**  **(N=10)** | **Total**  **(N=18)** |
| --- | --- | --- | --- |
| **Evaluable for response**^†^ | **6** | **6** | **12** |
| **Response**,^‡^ n (%) |  |  |  |
| Complete response | 0 | 1 (17) | 1 (8) |
| Very good partial response | 1 (17) | 0 | 1 (8) |
| Partial response | 3 (50) | 1 (17) | 4 (33) |
| Minimal response | 1 (17) | 0 | 1 (8) |
| Stable disease | 0 | 3 (50) | 3 (25) |
| Progressive disease | 1 (17) | 1 (17) | 2 (17) |

^†^ Response data was not available for all patients, therefore response analysis was performed on 12 total patients (Sd =6 ; SVd = 6)

^‡^ Percentages based on number of patients evaluable for response

Sd = selinexor and dexamethasone; SVd = selinexor, bortezomib, and dexamethasone

**Table S2. Patients with Disease Progression and Death (Events in the Survival Analysis) and Patients with Events Censored Along with Reasons for Censoring**

|  | **All Patients (N=18)** | **Sd  (N=8)** | **SVd  (N=10)** | **Patients with Partial Response or Better (N=6)** |
| --- | --- | --- | --- | --- |
| **Patients with Events**, n (%) | 13 (72.2) | 5 (62.5) | 8 (80.0) | 4 (66.7) |
| Death | 1 (5.6) | 1 (12.5) | 0 | 0 |
| Disease progression | 12 (66.7) | 4 (50.0) | 8 (80.0) | 4 (66.7) |
| **Patient Events Censored**, n (%) | 5 (27.8) | 3 (37.5) | 2 (20.0) | 2 (33.3) |
| COVID infection | 1 (5.6) | 1 (12.5) | 0 | 0 |
| Discontinued due to intolerance | 1 (5.6) | 1 (12.5) | 0 | 1 (16.7) |
| Patient decision to discontinue | 1 (5.6) | 1 (12.5) | 0 | 1 (16.7) |
| Patient had transplant | 1 (5.6) | 0 | 1 (10.0) | 0 |
| Patient unable to tolerate the treatment | 1 (5.6) | 0 | 1 (10.0) | 0 |

**S**d = selinexor and dexamethasone; **S**Vd = selinexor, bortezomib, and dexamethasone

**Table S3. Adverse Events by Overall and by Severity**

|  | **Sd**  **(N=8)** | | | **SVd (N=10)** | | |
| --- | --- | --- | --- | --- | --- | --- |
|  | **Grade 1-2**  **n (%)** | **Grade ≥ 3 n (%)** | **Total**  **n (%)** | **Grade 1-2**  **n (%)** | **Grade ≥ 3 n (%)** | **Total**  **n (%)** |
| **Hematologic AEs** | | | | | | |
| Thrombocytopenia | 0 | 4 (50) | 4 (50) | 2 (20) | 4 (40) | 6 (60) |
| Neutropenia | 0 | 2 (25) | 2 (25) | 2 (20) | 0 | 2 (20) |
| Cytopenia | 0 | 1 (13) | 1 (13) | 0 | 0 | 0 |
| Pancytopenia | 0 | 0 | 0 | 1 (10) | 0 | 1 (10) |
| **Non- Hematologic AEs** | | | | | | |
| Anorexia | 0 | 1 (13) | 1 (13) | 0 | 1 (10) | 1 (10) |
| Fatigue | 0 | 0 | 0 | 0 | 1 (10) | 1 (10) |
| Nausea | 1 (13) | 2 (25) | 3 (38) | 2 (20) | 2 (20) | 4 (40) |
| Weight loss | 0 | 1 (13) | 1 (13) | 0 | 0 | 0 |
| Personality change | 0 | 1 (13) | 1 (13) | 0 | 0 | 0 |
| Hyponatremia | 0 | 1 (13) | 1 (13) | 3 (30) | 0 | 3 (30) |
| Decreased appetite | 1 (13) | 0 | 1 (13) | 1 (10) | 0 | 1 (10) |
| Sepsis | 0 | 1 (13) | 1 (13) | 0 | 0 | 0 |
| Seizure | 0 | 1 (13) | 1 (13) | 0 | 0 | 0 |
| Diarrhea | 1 (13) | 0 | 1 (13) | 1 (10) | 0 | 1 (10) |
| Vomiting | 0 | 0 | 0 | 1 (10) | 2 (20) | 3 (30) |
| Somnolence | 1 (13) | 0 | 1 (13) | 0 | 0 | 0 |
| Acute kidney injury | 0 | 2 (25) | 2 (25) | 0 | 0 | 0 |
| Pneumonia | 0 | 2 (25) | 2 (25) | 0 | 0 | 0 |
| COVID-19 | 0 | 1 (13) | 1 (13) | 0 | 0 | 0 |
| Enterocolitis hemorrhagic | 0 | 0 | 0 | 1 (10) | 0 | 1 (10) |
| Dizziness | 0 | 0 | 0 | 1 (10) | 0 | 1 (10) |
| Malaise | 0 | 0 | 0 | 1 (10) | 0 | 1 (10) |
| Urinary tract infection | 0 | 0 | 0 | 1 (10) | 0 | 1 (10) |
| Abnormal liver function test | 0 | 0 | 0 | 1 (10) | 0 | 1 (10) |
| Overdose | 0 | 0 | 0 | 0 | 1 (10) | 1 (10) |

AE = adverse event; **S**d = selinexor and dexamethasone; **S**Vd = selinexor, bortezomib, and dexamethasone
